# Validation of self-reported morbidities in the Korean Atomic Bomb Survivor Cohort

**DOI:** 10.4178/epih.e2024058

**Published:** 2024-06-28

**Authors:** Ansun Jeong, Somin Jeon, Seong-geun Moon, Mi Kyung Kim, Inah Kim, Yu-Mi Kim, Boyoung Park

**Affiliations:** 1 Department of Health Sciences, Hanyang University, Seoul, Korea; 2 Department of Preventive Medicine, Hanyang University College of Medicine, Seoul, Korea; 3 Department of Occupational and Environmental Medicine, Hanyang University College of Medicine, Seoul, Korea; 4 Institute of Bioscience and Biotechnology, Hanyang University, Seoul, Korea

**Keywords:** Validation, Self-report, Questionnaires, Medical record, Morbidities, Atomic bomb

## Abstract

**OBJECTIVES:**

This study aimed to evaluate the agreement of disease status collected through a survey of the Korean Atomic Bomb Survivor Cohort (K-ABC), compared with medical claim records from the Korean National Health Insurance Service (NHIS) database and the Korean Central Cancer Registry (KCCR).

**METHODS:**

Data on the lifetime physician-diagnosed morbidities of 1,215 K-ABC participants were collected through an interviewer-administered questionnaire between 2020 and 2022. Survey data were linked to the NHIS and KCCR databases. Eleven diseases were included for validation. We evaluated the following indicators: sensitivity, specificity, positive predictive value, negative predictive value, accuracy, the area under the curve, and the kappa coefficient.

**RESULTS:**

The mean±standard deviation age was 62.1±18.7 years, and 42.6% of the participants were aged ≥70 years. Hypertension and cataracts showed the highest prevalence rates (33.8 and 28.8%, respectively). Hypertension, diabetes, and cancer demonstrated high sensitivity (>0.8) and specificity (>0.9), whereas diabetes, cancer, myocardial infarction, angina pectoris, and asthma exhibited high accuracy (>0.9). In contrast, arthritis, allergic rhinitis, and asthma showed low sensitivity (<0.4) and kappa values (<0.3). In the participants aged ≥70 years, the kappa value was ≥0.4 for all diseases except arthritis, allergic rhinitis, and asthma.

**CONCLUSIONS:**

The results from this initial analysis showed relatively high agreement between the survey and NHIS/KCCR databases, especially for hypertension, diabetes, and cancer. Our findings suggest that the information on morbidities collected through the questionnaires in this cohort was valid for both younger and older individuals.

## INTRODUCTION

The atomic bombings of Hiroshima and Nagasaki in 1945 affected not only Japanese individuals but also approximately 100,000 Koreans. Approximately 50,000 of these Koreans returned to Korea after liberation [1]. In 2020, the Korean Atomic Bomb Survivor Cohort (K-ABC) study was established to examine the health status of these survivors and the transgenerational health effects on their descendants. The methodology of the K-ABC study has been detailed in previous publications [2].

Epidemiological studies have employed various methods such as face-to-face, telephone, and mail surveys to assess participants’ health status [3]. Numerous cohort studies have also utilized surveys to monitor changes in health status and the progression of diseases over a follow-up period [4-6]. However, the morbidity or incidence rates of diseases reported through survey questionnaires may be inaccurate for several reasons, including memory decay, misunderstandings, or a reluctance to report [7-9]. Therefore, it is important to confirm the validity of disease status or progression as measured by survey questionnaires to ensure the reliability of epidemiological study results.

A commonly used method to assess the accuracy of reported disease prevalence or incidence involves comparing self-reported diseases with medical records or claims data [9,10]. Research on the validity of self-reported diseases, in terms of their agreement with medical records or claims data, has been conducted across various groups. These include participants in specific studies [4-6] and specific populations such as older adults, disabled older female, individuals in managed care, or those with severe morbidities [10-12]. Considering the difficult life and health experiences of Korean atomic bomb survivors over many years since their exposure, it is crucial to evaluate the validity of self-reported morbidities in these groups. Furthermore, the K-ABC study encompasses a wide age range among its participants, necessitating an age-stratified assessment of the validity of self-reported morbidities.

Therefore, this study aimed to evaluate the agreement between self-reported disease morbidities collected through face-to-face interviews with participants of the K-ABC and medical claims data from the Korean National Health Insurance Service (NHIS) database and the Korean Central Cancer Registry (KCCR). Additionally, the agreement was assessed and stratified by age group to determine if self-reporting accuracy was lower among the elderly population.

## MATERIALS AND METHODS

### Study population

The eligibility criteria for the K-ABC study included Korean atomic bomb survivors (first generation), their children (second generation), and their grandchildren (third generation) [2]. The first generation is defined under the Korean Victims of Atomic Bomb Special Act as individuals who were in Hiroshima or Nagasaki, Japan, at the time of the atomic bomb detonations, those within 3.5 km of the hypocenter within two weeks following the bombings, individuals engaged in relief activities post-bombing, those *in utero* at the time of the bombing, and individuals registered with the Korean Red Cross as atomic bomb victims [2,13]. The second generation consists of children of the first generation, and the third generation consists of grandchildren of the first generation [2,13]. Between 2020 and 2022, out of 1,251 individuals who provided informed consent to participate in the survey, 28 declined to complete the questionnaire due to health issues or other personal reasons. Thus, 1,223 participants completed the survey questionnaire and constituted the target population for data linkage.

### Measurement of morbidity status using the survey questionnaire

Trained interviewers conducted standardized questionnaire-based face-to-face interviews at clinics participating in the K-ABC study. To ascertain morbidity status, participants were asked, “Have you ever been diagnosed with [a disease] by a physician during your lifetime?” A “yes” response indicated the presence of disease morbidity, while a “no, I have not” response indicated its absence. This study assessed and validated the following 11 diseases: hypertension, diabetes, cancer, myocardial infarction/angina pectoris (MI/AP), osteoporosis, arthritis, thyroid disease, allergic rhinitis, asthma, cataract, and prostate disease.

### Validation data source: the National Health Information Database and Korea Central Cancer Registry

Two datasets were used to validate self-reported morbidities. The NHIS, which is the sole medical insurer in Korea, provides mandatory health insurance to the entire population. In 2011, the NHIS established the National Health Information Database (NHID) specifically for research purposes. This database contains all claim records from clinics and hospitals dating back to 2002 [14]. Using the resident registration number, individual survey data were linked to the NHIS claims data spanning from 2002 to 2020, the earliest and latest data available at the time of analysis.

**Figure 1.**
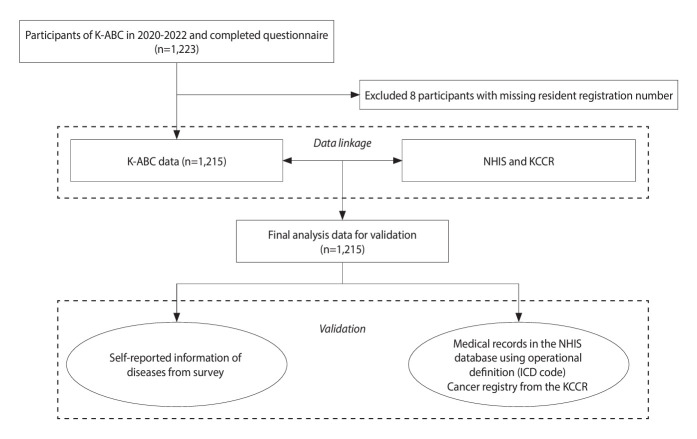
Flow diagram of the data linkage process between the Korean Atomic Bomb Survivor Cohort (K-ABC) and National Health Insurance Service (NHIS) and Korea Central Cancer Registry (KCCR). ICD-10, International Classification of Diseases, 10th revision.

The KCCR is a population-based cancer registry that covers > 97% of all new cancer cases in Korea. Since 1999, the KCCR has annually reported nationwide cancer statistics [15]. For cancer validation, we additionally linked individual reports of cancer morbidity with the Korea National Cancer Incidence Database (KNCI DB), which is reported by the KCCR. Since the last available KNCI DB was 2020, the KNCI DB between 1999 and 2020 was utilized. The process of data linkage between the atomic bomb survivor cohort and NHIS and KCCR data is shown in Figure 1. Of the 1,223 K-ABC participants who completed the survey in 2020-2022, 8 with missing resident registration numbers were excluded, leaving 1,215 participants in the analysis. We analyzed the data in 2023.

### Operational definition of disease morbidities from the National Health Information Database

The operational definitions of diseases in this study were based on the International Classification of Diseases, 10th revision (ICD-10) diagnosis codes provided by the NHID. These definitions drew on disease characteristics and previous research utilizing the NHIS database. For instance, cancer was defined using a combination of ICD-10 codes C00-D48 and special intractable disease codes specific to cancer patients in the Korean healthcare system [16]. For other diseases, the criteria included the number of hospital visits with relevant ICD-10 codes as the primary diagnostic code, tailored to the disease characteristics. For example, chronic conditions such as hypertension and diabetes, which necessitate regular check-ups for medication management, were defined by at least 3 visits within 1 year [17,18]. MI/AP was characterized by more than two days of hospitalization, aligning with definitions from previous studies [19,20]. The operational definition of each disease, based on the claims data with the primary diagnosis ICD-10 code, is presented in Supplementary Material 1.

### Statistical analysis

Baseline characteristics of the K-ABC participants were presented as means and proportions. To assess the agreement between self-reported morbidity status and the NHIS operational definition, with KCCR serving as the standard reference, we utilized the following measures: sensitivity, specificity, positive predictive value (PPV), negative predictive value (NPV), accuracy, and area under the curve (AUC). Sensitivity is the ability of a test to accurately identify individuals with a specific disease, while specificity is the ability of a test to correctly recognize individuals who do not have the disease of interest [21,22]. The PPV indicates the proportion of individuals who test positive and actually have the condition of interest, whereas the NPV indicates the proportion of individuals who test negative and are truly free of the condition [21,22]. Accuracy reflects the proportion of participants correctly classified (true positives and true negatives) out of the total number of participants [22]. The AUC, used in conjunction with the receiver operating characteristic (ROC) curve, evaluates classification tasks by considering both sensitivity and specificity, which represent trade-offs. The kappa coefficient, which measures the degree of agreement beyond what would be expected by chance, is valuable for assessing reliability [22,23]. We calculated sensitivity, specificity, PPV, NPV, accuracy, AUC, and the kappa coefficient, stratified by participants aged < 70 years and ≥ 70 years.

### Ethics statement

This study received approval from the Institutional Review Board of Hanyang University (approval No. HYUIRB-202007-014-17).

## RESULTS

### Baseline characteristics of atomic bomb survivors

The general characteristics of the 1,215 K-ABC participants recruited between 2020 and 2022 are presented in Table 1. The mean age of the participants was 62.1 years (47.6% male and 52.4% female). Among the participants, 31.6% had a monthly average household income of < 1 million Korean won and 57.0% were married. Furthermore, 41.8% of the participants had a middle school education or lower, and 61.8% had never smoked. Of the 1,215 participants, 451, 631, and 133 were first-generation, second-generation, and third-generation atomic bomb survivors, respectively.

### Prevalence of diseases among atomic bomb survivors

Based on a self-reported questionnaire, the disease with the highest prevalence among the K-ABC participants was hypertension (33.8%), followed by cataracts (28.8%), prostate disease (26.1%), and arthritis (22.7%) (Table 2). Based on the medical claims data with operational definitions of the NHIS, hypertension was the most common morbidity in the K-ABC participants (34.4%), followed by cataracts (31.9%), prostate disease (31.8%), and arthritis (20.7%).

### Agreement of self-reported morbidity compared with the claims data or cancer registry among the total participants

The overall sensitivity, specificity, PPV, NPV, accuracy, AUC, and kappa coefficient of the self-reported morbidities compared with the operational definition of claims data from the NHID are presented in Table 3. High accuracy (> 0.80) was observed for hypertension (0.89), diabetes (0.93), cancer (0.96 for both sources), MI/AP (0.95), osteoporosis (0.85), thyroid disease (0.89), asthma (0.91), cataracts (0.86), and prostate disease (0.82). Diseases with an accuracy of < 0.80 included arthritis (0.73) and allergic rhinitis (0.78). For all diseases, high specificity (> 0.80) was observed, but the sensitivity varied between 0.25 and 0.86. The AUCs for 5 diseases (hypertension, diabetes, cancer, cataracts, and prostate disease) showed very good or higher levels of accuracy (> 0.80). Other diseases showed a good level of accuracy (AUC of 0.7-0.8). However, the AUCs for arthritis, allergic rhinitis, and asthma were approximately ≤ 0.6.

The highest kappa statistic was observed for cancer, with nearly perfect agreement (κ=0.81). Substantial agreement (κ=0.6 to 0.8) was observed for hypertension (κ=0.76), diabetes (κ=0.75), and cataracts (κ=0.66). Moderate agreement (κ=0.4 to 0.8) was presented for MI/AP (κ=0.48), osteoporosis (κ=0.45), thyroid disease (κ=0.45) and prostate disease (κ=0.57). Three diseases showed poor to fair agreement: arthritis (κ=0.19), allergic rhinitis (κ=0.16), and asthma (κ=0.26).

### Agreement of self-reported morbidity compared with the claims data or cancer registry by age group

The results of the validity of self-reported morbidities compared with the claims data of the participants aged < 70 years and ≥ 70 years are presented in Table 4, respectively. For most diseases, the participants aged < 70 years and ≥ 70 years showed similar validity regarding AUCs and reliability in terms of the kappa statistics. However, the kappa statistics for arthritis, allergic rhinitis, and asthma were lower in both age groups, and individuals aged ≥ 70 years showed lower kappa coefficients compared with those aged < 70 years. Otherwise, individuals aged ≥ 70 years showed better AUCs and kappa coefficients for osteoporosis and prostate disease.

## DISCUSSION

This study evaluated the agreement between self-reported chronic diseases diagnosed by clinicians and medical claims or cancer registry data among Korean atomic bomb survivors as a surrogate index of the validity of self-reported morbidities. In our study, hypertension, diabetes, cancer, and cataracts showed high levels of overall accuracy, AUCs, and agreement between self-reported morbidities and medical claims/registry data. For MI/AP, osteoporosis, thyroid disease, and prostate disease, the self-reported information and medical claims data showed particularly high levels of accuracy (accuracy > 0.80; AUC > 0.70) and a moderate level of agreement (κ=0.40 to 0.60). High levels of accuracy and agreement for such as hypertension, diabetes, and cancer were consistently observed in both individuals aged < 70 years and ≥ 70 years. Therefore, self-reported morbidities are an effective measure for assessing these diseases in general and elderly populations. The low agreement for arthritis, allergic rhinitis, and asthma may be substantially imprecise when relying on self-reports.

Our study observed high accuracy and agreement between self-reported and medical claims data in diabetes and cancer diseases, similar to previous studies [11,12,24,25]. These diseases require intensive medical treatment and lifestyle modifications, impacting not only the patients’ quality of life but also that of their family members, thereby increasing awareness [26,27]. In Korea, the diabetes awareness rate—defined as the percentage of individuals diagnosed with diabetes by a doctor among those with the disease—was 66.6%, with 62.4% being treated with glucose-lowering agents or insulin [28]. Regarding cancer, the high awareness—with correspondingly high levels of accuracy and agreement—can be attributed to nationwide registries covering > 97% of new cancer cases in Korea and comprehensive national cancer control programs [15,29]. A Health Examinees study also showed a high sensitivity and PPV for self-reported overall cancer history [30]. Similarly, the accuracy and agreement of cancer data based on self-reports, when compared with two reference standards (NHID and KCCR), confirmed the high completeness of both the claims data and the registry, as indicated by previous research [16].

A meta-analysis reported that self-reported hypertension had low sensitivity and high specificity [31], whereas this condition exhibited not only high sensitivity and specificity (> 0.80), but also a high kappa coefficient, in this study population. Limited access to healthcare services, followed by low awareness, was suggested as a cause of the low pooled sensitivity of 0.24 in the meta-analysis [31]. In Korea, the high accessibility of healthcare services, including nationwide covered medical insurance services and chronic disease management programs [32], could have contributed to the high accuracy and agreement of self-reported hypertension. The awareness rate of hypertension was reported to be 71.2%, and 67.0% of individuals with hypertension were reported to take antihypertensive medication [28].

Previous studies have demonstrated a low level of agreement or accuracy in self-reported cataracts when compared to medical records, even among individuals experiencing vision loss [33-35]. However, the accuracy of self-reported cataract surgery was high in both the elderly and general populations [34,36]. Despite the poor recognition of cataracts among affected individuals in Korea, there was a high level of agreement between self-reported cataracts diagnosed by physicians and medical claims in this group. In Korea, cataract operations are the most frequently performed surgical procedures, with 945 operations per 100,000 persons recorded in 2015, and the annual number of these procedures has been increasing rapidly [37]. Another study showed that 7.0% of the Korean population aged ≥ 40 years had undergone cataract surgery, while this proportion reached nearly 50% among adults aged ≥ 80 years [38]. The high frequency of cataract surgery may contribute to the increased accuracy and agreement between self-reported clinician diagnoses and medical claims. However, when considering treatments not covered by insurance or those covered by private insurance for minor chronic diseases, combining disease codes with treatments could reduce the accuracy of disease identification. Therefore, we included disease codes and hospital visits within a 1-year period, anticipating that this approach would encompass hospital visits for both treatment and diagnosis or follow-up.

Studies have shown high sensitivity, specificity, PPV, and kappa coefficients for self-reported acute myocardial infarction [25,39]. In our study, the sensitivity, PPV, and kappa coefficient for MI/AP were lower than in previous studies [25,39]. Another study among older disabled females showed high agreement for self-reported angina pectoris (κ=0.73) but lower agreement for other types of heart diseases, including myocardial infarction (κ< 0.5) [12]. Because our study surveyed MI/AP as a single item, the accuracy of each condition (i.e., myocardial infarction or angina pectoris) was diluted and showed moderate agreement. One study suggested that patients were confused about various cardiovascular diseases, including heart attack, coronary disease, and myocardial infarction, due to the relative unfamiliarity and intermittent duration of symptoms [40]. In another study focusing on various cancer types in Korea, prostate cancer had the highest PPV [30]. This could be due to patients’ better understanding of prostate-related diseases, which might explain the moderate agreement observed between self-reported prostate disease diagnoses and medical claims. The moderate level of agreement observed in this study for osteoporosis and thyroid disease was comparable to, or higher than, those reported in previous studies [12,41]. The broad disease spectrum encompassed by osteoporosis and various thyroid issues, including hyperthyroidism, hypothyroidism, and goiter, may account for the moderate agreement noted between these diseases.

Arthritis, allergic rhinitis, and asthma demonstrated low levels of accuracy and agreement in diagnosis. For arthritis, the literature shows a range of accuracy and agreement levels, some of which are better than [42,43] or similar to our results [12,44]. The confusion surrounding arthritis may stem from its various forms, such as osteoarthritis, rheumatoid arthritis, and polyarthritis. Notably, osteoarthritis has multiple definitions and typically does not severely impact daily activities [45,46]. Allergic rhinitis and asthma are often mistaken for conditions like sinusitis and upper respiratory infections due to overlapping symptoms [47]. This confusion likely contributes to the low accuracy and agreement observed in diagnosing these diseases.

When comparing accuracy and agreement among individuals aged under 70 and those 70 or older, similar levels of accuracy and agreement were observed in both older and younger groups. Research on the accuracy of self-reported diseases in older populations has demonstrated high validity for several conditions, including diabetes, cancer, hip fracture, stroke, disc diseases, thyroid dysfunction, and asthma/chronic obstructive pulmonary disease, although there are variations across studies [11,12,44]. Therefore, while the accuracy of self-reported diseases may vary depending on the characteristics of the disease, the impact of the disease itself is likely minimal. For conditions such as osteoporosis and prostate disease, participants aged ≥ 70 years exhibited higher AUCs and kappa coefficients than those < 70, which is likely attributable to the increased prevalence of these diseases with age [48,49].

This study has certain limitations. First, the disease information reported in our survey reflects lifetime experiences, whereas the NHIS claims data only covers the period from 2002 to 2020, and the KCCR data includes cancer registrations from 1999 to 2020. This discrepancy in time frames between the self-reported data and the claims or registry data may have resulted in an underestimation of the diseases recorded in the NHIS database. Despite this, the accuracy of the data remains significantly high for conditions such as hypertension, cancer, diabetes, and myocardial infarction. Second, our use of an operational definition based on the NHIS dataset, which is based solely on the number of healthcare provider visits for a specific disease, is a common approach in studies validating survey data. However, in some instances, a disease definition that relies on the number of visits might also consider prescriptions [50]. Although this operational definition of claims data has potential limitations, it primarily reflects the diagnosis made by the examining physician. Therefore, the disease code assigned is likely the one the doctor most strongly suspected.

This study established data on the accuracy of self-reported morbidities for most diseases in the K-ABC, particularly for conditions such as hypertension, diabetes, cancer, and cataracts, which require regular or intensive treatment. However, there was less agreement on diseases treated based on the occurrence of symptoms, including arthritis, allergic rhinitis, and asthma. Therefore, the characteristics of each disease should be taken into account during the collection of self-reported morbidity data.

## Figures and Tables

**Table 1.** Characteristics of the K-ABC participants (n=1,215)

Characteristics	K-ABC (n=1,215)
Age, mean±SD (yr)	62.1±18.7
<39	122 (10.0)
40-49	149 (12.3)
50-59	250 (20.6)
60-69	177 (14.6)
70-79	279 (23.0)
≥80	238 (19.6)
Sex	
Male	578 (47.6)
Female	637 (52.4)
BMI, mean±SD (kg/m^2^)	24.4±3.9
Average household income/mo (1 million KRW)	
<1	384 (31.6)
1 to <3	295 (24.3)
≥3	395 (32.5)
Missing	141 (11.6)
Marital status	
Married	693 (57.0)
Widowed/Divorced	231 (19.0)
Unmarried	176 (14.5)
Missing	1 (0.1)
Education	
≤Middle school	508 (41.8)
High school	366 (30.1)
≥College	339 (27.9)
Missing	2 (0.2)
Smoking	
Non-smoker	751 (61.8)
Ex-smoker	169 (13.9)
Current	275 (22.6)
Missing	20 (1.7)
Alcohol consumption	
Non-drinker	537 (44.2)
Ex-drinker	142 (11.7)
Current	531 (43.7)
Missing	5 (0.4)
Atomic Bomb Survivor generation^1^	
First	451 (37.1)
Second	631 (51.9)
Third	133 (11.0)

Values are presented as number (%). K-ABC, Korean Atomic Bomb Survivor Cohort; SD, standard deviation; BMI, body mass index; KRW, Korean won.

1 The first generation experienced atomic bomb damage in 1945, while the second generation referred to their children and the third generation to their grandchildren.

**Table 2.** Comparison of disease prevalence between self-report questionnaires and medical record history (n=1,215)

Diseases	Questionnaire (survey)		Medical records (NHIS)	
	No. of diagnoses	Prevalence	No. of diagnoses	Prevalence
Hypertension	411	33.8	418	34.4
Diabetes	214	17.6	198	16.3
Cancer (NHIS)^1^	133	10.9	126	10.4
Cancer (KCCR)^2^	133	10.9	125	10.3
MI/AP	73	6.0	61	5.0
Osteoporosis	209	17.2	196	16.1
Arthritis	276	22.7	251	20.7
Thyroid disease	141	11.6	122	10.0
Allergic rhinitis	171	14.1	210	17.3
Asthma	61	5.0	91	7.5
Cataracts	350	28.8	388	31.9
Prostate disease^3^	151	26.1	184	31.8

NHIS, National Health Insurance Service; KCCR, Korea Central Cancer Registry; MI/AP, myocardial infarction/angina pectoris.

1 Cancer definition using the primary diagnosis from the NHIS claims database.

2 Cancer definition using the national cancer registration database.

3 The prevalence of prostate disease was calculated in males.

**Table 3.** Validation results of the operational definition of chronic diseases based on the K-ABC data compared to the NHIS criteria as a standard reference

Diseases	TP	FP	FN	TN	Accuracy	Sensitivity	Specificity	AUC	PPV	NPV	Kappa
Hypertension	348	63	70	734	0.89 (0.87, 0.91)	0.83 (0.80, 0.87)	0.92 (0.90, 0.94)	0.88 (0.86, 0.90)	0.85 (0.81, 0.88)	0.91 (0.89, 0.93)	0.76 (0.72, 0.80)
Diabetes	164	50	34	967	0.93 (0.92, 0.94)	0.83 (0.78, 0.88)	0.95 (0.94, 0.96)	0.87 (0.84, 0.90)	0.77 (0.71, 0.82)	0.97 (0.95, 0.98)	0.75 (0.70, 0.80)
Cancer (NHIS)^1^	107	26	19	1,063	0.96 (0.95, 0.97)	0.85 (0.79, 0.91)	0.98 (0.97, 0.99)	0.89 (0.86, 0.93)	0.80 (0.74, 0.87)	0.98 (0.97, 0.99)	0.81 (0.75, 0.86)
Cancer (KCCR)^2^	107	26	18	1,064	0.96 (0.95, 0.97)	0.86 (0.79, 0.92)	0.98 (0.97, 0.99)	0.89 (0.86, 0.93)	0.80 (0.73, 0.87)	0.98 (0.98, 0.99)	0.81 (0.75, 0.86)
MI/AP	34	39	27	1,115	0.95 (0.93, 0.96)	0.56 (0.43, 0.68)	0.97 (0.96, 0.98)	0.72 (0.66, 0.78)	0.47 (0.35, 0.58)	0.98 (0.97, 0.99)	0.48 (0.37, 0.59)
Osteoporosis	110	99	86	920	0.85 (0.83, 0.87)	0.56 (0.49, 0.63)	0.90 (0.88, 0.92)	0.72 (0.69, 0.76)	0.53 (0.46, 0.59)	0.91 (0.90, 0.93)	0.45 (0.39, 0.52)
Arthritis	97	179	154	785	0.73 (0.70, 0.75)	0.39 (0.33, 0.45)	0.81 (0.79, 0.84)	0.59 (0.56, 0.62)	0.35 (0.30, 0.41)	0.84 (0.81, 0.86)	0.19 (0.13, 0.26)
Thyroid disease	67	74	55	1,019	0.89 (0.88, 0.91)	0.55 (0.46, 0.64)	0.93 (0.92, 0.95)	0.71 (0.67, 0.75)	0.48 (0.39, 0.56)	0.95 (0.94, 0.96)	0.45 (0.37, 0.53)
Allergic rhinitis	55	116	155	889	0.78 (0.75, 0.80)	0.26 (0.20, 0.32)	0.88 (0.86, 0.90)	0.59 (0.55, 0.62)	0.32 (0.25, 0.39)	0.85 (0.83, 0.87)	0.16 (0.09, 0.22)
Asthma	23	38	68	1,086	0.91 (0.90, 0.93)	0.25 (0.16, 0.34)	0.97 (0.96, 0.98)	0.66 (0.60, 0.72)	0.38 (0.26, 0.50)	0.94 (0.93, 0.95)	0.26 (0.16, 0.36)
Cataracts	282	68	106	759	0.86 (0.84, 0.88)	0.73 (0.68, 0.77)	0.92 (0.90, 0.94)	0.84 (0.82, 0.87)	0.81 (0.76, 0.85)	0.88 (0.86, 0.90)	0.66 (0.62, 0.71)
Prostate disease	116	35	68	359	0.82 (0.79, 0.85)	0.63 (0.56, 0.70)	0.91 (0.88, 0.94)	0.80 (0.77, 0.84)	0.77 (0.70, 0.84)	0.84 (0.81, 0.88)	0.57 (0.50, 0.64)

Values are presented as point estimate (95% confidence interval). K-ABC, Korean Atomic Bomb Survivor Cohort; NHIS, National Health Insurance Service; TP, true positive; FP, false positive; TN, true negative; FN, false negative; PPV, positive predictive value; NPV, negative predictive value; AUC, area under the curve; KCCR, Korea Central Cancer Registry; MI/AP, myocardial infarction/angina pectoris.

1 Cancer definition using the primary diagnosis from the NHIS claims database.

2 Cancer definition using the national cancer registration database.

**Table 4.** The validation results of the operational definition of chronic diseases among participants aged <70 and those aged ≥70 in the K-ABC data were compared to the NHIS criteria as a standard reference

Diseases	TP	FP	FN	TN	Accuracy	Sensitivity	Specificity	AUC	PPV	NPV	Kappa
Age <70											
Hypertension	95	33	13	557	0.93 (0.91, 0.95)	0.88 (0.82, 0.94)	0.94 (0.93, 0.96)	0.86 (0.82, 0.90)	0.74 (0.67, 0.82)	0.98 (0.96, 0.99)	0.77 (0.70, 0.83)
Diabetes	51	23	9	615	0.95 (0.94, 0.97)	0.85 (0.76, 0.94)	0.96 (0.95, 0.98)	0.84 (0.78, 0.89)	0.69 (0.58, 0.79)	0.99 (0.98, 0.99)	0.74 (0.65, 0.82)
Cancer (NHIS)^1^	36	12	1	649	0.98 (0.97, 0.99)	0.97 (0.92, 1.00)	0.98 (0.97, 0.99)	0.90 (0.86, 0.94)	0.75 (0.63, 0.87)	1.00 (1.00, 1.00)	0.84 (0.75, 0.92)
Cancer (KCCR)^2^	37	11	1	649	0.98 (0.97, 0.99)	0.97 (0.92, 1.00)	0.98 (0.97, 0.99)	0.88 (0.82, 0.94)	0.77 (0.65, 0.89)	1.00 (1.00, 1.00)	0.85 (0.77, 0.93)
MI/AP	6	7	1	684	0.99 (0.98, 1.00)	0.86 (0.60, 1.00)	0.99 (0.98, 1.00)	0.73 (0.59, 0.87)	0.46 (0.19, 0.73)	1.00 (1.00, 1.00)	0.59 (0.34, 0.85)
Osteoporosis	10	41	7	640	0.93 (0.91, 0.95)	0.59 (0.35, 0.82)	0.94 (0.92, 0.96)	0.59 (0.54, 0.65)	0.20 (0.09, 0.31)	0.99 (0.98, 1.00)	0.27 (0.13, 0.41)
Arthritis	20	80	40	558	0.83 (0.80, 0.86)	0.33 (0.21, 0.45)	0.87 (0.85, 0.90)	0.57 (0.53, 0.61)	0.20 (0.12, 0.28)	0.93 (0.91, 0.95)	0.16 (0.07, 0.25)
Thyroid disease	29	39	29	601	0.90 (0.88, 0.92)	0.50 (0.37, 0.63)	0.94 (0.92, 0.96)	0.69 (0.63, 0.75)	0.43 (0.31, 0.54)	0.95 (0.94, 0.97)	0.41 (0.29, 0.52)
Allergic rhinitis	42	96	58	502	0.78 (0.75, 0.81)	0.42 (0.32, 0.52)	0.84 (0.81, 0.87)	0.60 (0.56, 0.64)	0.30 (0.23, 0.38)	0.90 (0.87, 0.92)	0.22 (0.14, 0.31)
Asthma	11	17	21	649	0.95 (0.93, 0.96)	0.34 (0.18, 0.51)	0.97 (0.96, 0.99)	0.68 (0.59, 0.77)	0.39 (0.21, 0.57)	0.97 (0.96, 0.98)	0.34 (0.18, 0.50)
Cataracts	32	29	15	622	0.94 (0.92, 0.95)	0.68 (0.55, 0.81)	0.96 (0.94, 0.97)	0.75 (0.69, 0.81)	0.52 (0.40, 0.65)	0.98 (0.96, 0.99)	0.56 (0.44, 0.68)
Prostate disease	16	18	25	270	0.87 (0.83, 0.90)	0.39 (0.24, 0.54)	0.94 (0.91, 0.97)	0.69 (0.61, 0.78)	0.47 (0.30, 0.64)	0.92 (0.88, 0.95)	0.35 (0.20, 0.51)
Age ≥70											
Hypertension	253	30	57	177	0.83 (0.80, 0.86)	0.82 (0.77, 0.86)	0.86 (0.81, 0.90)	0.83 (0.79, 0.86)	0.89 (0.86, 0.93)	0.76 (0.70, 0.81)	0.66 (0.59, 0.72)
Diabetes	113	27	25	352	0.90 (0.87, 0.92)	0.82 (0.75, 0.88)	0.93 (0.90, 0.95)	0.87 (0.84,0.91)	0.81 (0.74, 0.87)	0.93 (0.91, 0.96)	0.74 (0.68, 0.81)
Cancer (NHIS)^1^	71	14	18	414	0.94 (0.91, 0.96)	0.80 (0.71, 0.88)	0.97 (0.95, 0.98)	0.90 (0.86, 0.94)	0.84 (0.76, 0.91)	0.96 (0.94, 0.98)	0.78 (0.71, 0.85)
Cancer (KCCR)^2^	70	15	17	415	0.94 (0.91, 0.96)	0.80 (0.72, 0.89)	0.97 (0.95, 0.98)	0.89 (0.85, 0.93)	0.82 (0.74, 0.90)	0.96 (0.94, 0.98)	0.78 (0.70, 0.85)
MI/AP	28	32	26	431	0.89 (0.86, 0.91)	0.52 (0.39, 0.65)	0.93 (0.91, 0.95)	0.70 (0.64, 0.77)	0.47 (0.34, 0.59)	0.94 (0.92, 0.96)	0.43 (0.31, 0.55)
Osteoporosis	100	58	79	280	0.74 (0.69, 0.77)	0.56 (0.49, 0.63)	0.83 (0.79, 0.87)	0.71 (0.66, 0.75)	0.63 (0.56, 0.71)	0.78 (0.74, 0.82)	0.40 (0.31, 0.48)
Arthritis	77	99	114	227	0.59 (0.54, 0.63)	0.40 (0.33, 0.47)	0.70 (0.65, 0.75)	0.55 (0.51, 0.60)	0.44 (0.36, 0.51)	0.67 (0.62, 0.72)	0.10 (0.01, 0.19)
Thyroid disease	38	35	26	418	0.88 (0.85, 0.91)	0.59 (0.47, 0.71)	0.92 (0.90, 0.95)	0.73 (0.67, 0.79)	0.52 (0.41, 0.64)	0.94 (0.92, 0.96)	0.49 (0.38, 0.60)
Allergic rhinitis	13	20	97	387	0.77 (0.74, 0.81)	0.12 (0.06, 0.18)	0.95 (0.93, 0.97)	0.60 (0.51, 0.68)	0.39 (0.23, 0.56)	0.80 (0.76, 0.84)	0.09 (0.01, 0.18)
Asthma	12	21	47	437	0.87 (0.84, 0.90)	0.20 (0.10, 0.31)	0.95 (0.94, 0.97)	0.63 (0.55, 0.72)	0.36 (0.20, 0.53)	0.90 (0.88, 0.93)	0.20 (0.07, 0.32)
Cataracts	250	39	91	137	0.75 (0.71, 0.79)	0.73 (0.69, 0.78)	0.78 (0.72, 0.84)	0.73 (0.70, 0.77)	0.87 (0.83, 0.90)	0.60 (0.54, 0.66)	0.48 (0.40, 0.55)
Prostate disease	100	17	43	89	0.76 (0.70, 0.81)	0.70 (0.62, 0.77)	0.84 (0.77, 0.91)	0.76 (0.71, 0.82)	0.85 (0.79, 0.92)	0.67 (0.59, 0.75)	0.52 (0.42, 0.63)

Values are presented as point estimate (95% confidence interval). K-ABC, Korean Atomic Bomb Survivor Cohort; NHIS, National Health Insurance Service; TP, true positive; FP, false positive; TN, true negative; FN, false negative; PPV, positive predictive value; NPV, negative predictive value; AUC, area under the curve; KCCR, Korea Central Cancer Registry; MI/AP, myocardial infarction/angina pectoris.

1 Cancer definition using the primary diagnosis from the NHIS claims database.

2 Cancer definition using the national cancer registration database.

## Supplementary materials

Supplementary Material 1. The definition of common diseases in Atomic Bomb Survivor Cohort data and the National Health Insurance Service (NHIS) data
