# Supplementary material for: Validation of self-reported morbidities in the Korean Atomic Bomb Survivor Cohort
Source: Epidemiol Health. 2024 Jun 28;46:e2024058. doi: 10.4178/epih.e2024058 (PMC11826016; doi:10.4178/epih.e2024058)
Supplement: Supplementary Material 1. — The definition of common diseases in Atomic Bomb Survivor Cohort data and the National Health Insurance Service (NHIS) data [file epih-46-e2024058-Supplementary-1.docx]

**Supplementary Material 1. The definition of common diseases in Atomic Bomb Survivor Cohort data and the National Health Insurance Service (NHIS) data**

| **Diseases** | **Code** | **ICD-10 codes and additional definitions** |
| --- | --- | --- |
| Hypertension | I10-I15 | Record of visiting the hospital three a year for hypertension |
| Diabetes | E11-E14 | Record of visiting the hospital three a year for diabetes |
| Cancer | C00-D48, V193-194 | Record of visiting the hospital at least once for cancer |
| Myocardial infarction/angina pectoris | I21-I22 | Record of hospitalization for myocardial infarction or angina pectoris for two days |
| Osteoporosis | M80-M82 | Record of visiting the hospital three a year for osteoporosis |
| Arthritis | M00-M03, M05-M09, M13 | Record of visiting the hospital four a year for arthritis |
| Thyroid disease | E00-E07 | Record of visiting the hospital four a year for thyroid disease |
| Allergic rhinitis | J30-J31 | Record of visiting the hospital four a year for allergic rhinitis |
| Asthma | J45-J46 | Record of visiting the hospital four a year for asthma |
| Cataract | H25-H28 | Record of visiting the hospital twice a year for cataract |
| Prostate disease | N40-N42 | Record of visiting the hospital four a year for prostate disease |
